# Resident vascular endothelial progenitor definition and function: the age of reckoning

**DOI:** 10.1007/s10456-021-09817-2

**Published:** 2021-09-09

**Authors:** James Dight, Jilai Zhao, Cassandra Styke, Kiarash Khosrotehrani, Jatin Patel

**Affiliations:** 1 The University of Queensland Diamantina Institute, 37 Kent Street, Woolloongabba, Brisbane, 4102 Australia; 2 Cancer and Ageing Research Program, School of Biomedical Sciences, Queensland University of Technology, 37 Kent Street, Woolloongabba, Brisbane, 4102 Australia

**Keywords:** Heterogeneity, Endothelium, Neovascularisation, Angiogenesis, Progenitors

## Abstract

The cardiovascular system is composed around the central function of the endothelium that lines the inner surfaces of its vessels. In recent years, the existence of a progenitor population within the endothelium has been validated through the study of endothelial colony-forming cells (ECFCs) in human peripheral blood and certain vascular beds. However, our knowledge on endothelial populations *in vivo* that can give rise to ECFCs in culture has been limited. In this review we report and analyse recent attempts at describing progenitor populations *in vivo* from murine studies that reflect the self-renewal and stemness capacity observed in ECFCs. We pinpoint seminal discoveries within the field, which have phenotypically defined, and functionally scrutinised these endothelial progenitors. Furthermore, we review recent publications utilising single-cell sequencing technologies to better understand the endothelium in homeostasis and pathology.

## Introduction

The cardiovascular system is essential in a number of key physiological processes such as the transport of oxygen and nutrients to tissues and providing the conduits for blood flow during the development and adult life [1]. The vascular endothelium, the intimal face of capillary, arterial, venous and lymphatic systems, is a partially permeable interface, which selectively permits the exchange of macromolecules and other cells alike into the tissue in sub-luminal spaces [2]. The endothelium is adaptive and can respond to both physiological and pathological stimuli including but not limited to ischaemia, oedema, shear stress, atherosclerotic plaque formation, wound healing or tumour formation. Whilst the endothelium can rapidly adapt to physiological or pathophysiological changes, the progressive degeneration caused by ageing and the continuous insult of environmental factors can significantly hinder the endothelium’s repair and maintenance mechanisms. To play such important and varying roles, endothelial cells (ECs) display a highly specialised and heterogeneous nature and their phenotypes vary between organs, within the same organ and even between neighbouring cells [3]. Although heterogeneous in nature, there are several defining characteristics of ECs. Firstly, their expression of tight junctions with cell–cell adhesive structures such as vascular endothelial (VE)-cadherin and secondly their intrinsic capacity for cellular turnover and migration [4]. The biological process of sprouting angiogenesis, whereby pre-existing vessels bifurcate to form new vessels is well established, yet literature describing the activation and potential of vascular stem cells in therapeutic and pathological contexts remains in a state of infancy. A recent and pivotal inflection point was reached in response to prominent publications highlighting the presence of tissue-resident vascular progenitors as essential drivers of adult neovascularisation [5]. This paradigm shift comes on the back of a group of findings reporting adult de novo vascularisation to be carried out independent of hematopoietic origins and elicited by tissue-resident endothelial progenitor cells (EPC). As is highlighted throughout this summation of recently defined EPCs, the diversity of these tissue-resident populations both phenotypically and functionally may be predominantly limited by organ specific and situational isolation of these cells. As new information comes to light in this growing field, we envision parallels of molecular and phenotypic characterisation of endothelial progenitors to emerge and accelerate cumulative research findings. With the advent of and improvement in mouse models, flow cytometry, gene expression and omics analysis coupled with emerging technologies, exciting opportunities near for the utilisation of endothelial progenitors for therapeutic and diagnostic purposes.

## Vascular network formation

The angiogenic process is fundamental in a myriad of biological processes including development, tissue repair and reproduction [6, 7]. Angiogenesis is closely governed by molecular pathways, which can instigate the rebuilding of vascular networks to support changes in tissue requirements [8–11]. Classical vascular network formation is understood to originate from sprouting angiogenesis that involves existing ECs detaching junctional adhesions and growing in the form of conditional tubes towards angiogenic signals [7, 11–14]. Hypoxic environments trigger local cellular production of vascular endothelial growth factor (VEGF), which activates quiescent endothelium to degrade nearby extracellular matrix (ECM) and activate a tip cell phenotype, whereby filopodia respond to VEGF-A gradients [10, 15]. Structural support is provided by the stalk cells just behind the tip cells as they elongate and reach a point which two tip cells meet and form an immature vascular lumen [9]. Reperfusion and blood flow into the newly formed vasculature increases oxygenation, downregulating VEGF production, followed by vessel stabilisation through pericyte migration and maturation [16].

A lesser understood and more controversial topic of interest is the body of research implicating EPC populations. The influential paper published by Asahara et al. in 1997 first illustrated the process of adult neovasculogenesis in an ischaemic mouse model [17]. The described putative EPCs were of human bone marrow origin and demonstrated acquisition of EC surface phenotype during in vitro culture. These cells also demonstrated colony-forming capacity and homing capabilities in vivo. Subsequent studies would go on to scrutinise and clarify that these putative EPCs could not illicit a lineage fate switch from hematopoietic origins nor could they integrate into the intimal layer [18] (reviewed by Yoder et al. [19]). Since this seminal discovery, stringent criteria have emerged to define what constitutes a true EPC. Cells that promote angiogenesis, including myeloid populations and mesenchymal stem/stromal cells, have previously been labelled as EPCs despite an inability to form endothelial layers of vessels de novo [20, 21]. Coincidentally, hematopoietic cells and vascular ECs share a suite of cell surface markers, leading to mislabelling of cells as an ‘*EPC*’ and consequently resulting in mixed findings [22, 23]. Thus, a recent consensus review has demarcated that EPCs must (1) have the ability to self-renew, (2) form blood vessels and (3) constitute the intimal layer as well as (4) having the capacity to be serially transplanted to form/incorporate into host vasculature [5, 19]. Consequently, hematopoietic cells previously labelled as EPCs have been re-established as myeloid angiogenic cells (MACs). Although this consensus has aided the field in providing some clarity to defining EPCs, the lineage and possible maturation hierarchy governed by a true endothelial progenitor population into mature endothelium remain controversial [17, 22, 24].

## Defining human endothelial progenitor cells

Considerable controversy has surfaced surrounding the identification, purification and clonal expansion of EPC and daughter endothelial populations in vitro. Typical endothelial phenotypes have been identified using a combination of CD31 (*PECAM1*), acetylated low-density lipoprotein (Ac-LDL) uptake and lectin (UEA-1) binding. However, studies have demonstrated that hematopoietic cells have the potential to acquire the CD31 antigen via passive transfer [25]. Furthermore, some monocyte populations are known to express the prominent endothelial markers CD31 and vascular endothelial growth factor receptor 2 (VEGFR2) complicating EPC isolation [26].

Traditionally, acquiring human EPCs has been conducted through isolating mononuclear CD34 + VERGFR2 + cells. Two such markers, however, routinely capture mature ECs, thus research groups have previously included CD133 for progenitor isolation [27–29]. Isolation strategies using CD133 + CD34 + VEGFR2 + cells have remained inconclusive, with some groups demonstrating isolation of ECs using this technique and others uncovering cells of hematopoietic origin and fate [22, 29]. To date, various studies have claimed to therapeutically administer EPCs, specifically in cases of myocardial infarction, limb ischaemia and tumour growth and observed integration into host vasculature [30–32]. However, other studies have claimed the contrary, suggesting EPC’s of these aforementioned cell surface phenotypes do not incorporate into the host vasculature [18, 33, 34]. A study by Medina et al. uncovered that MACs (previously mislabelled EPCs) were in fact molecularly and phenotypically distinct when compared to endothelial colony-forming cells (ECFCs), originally coined as outgrowth endothelial cells (OECs) [35]. Hematopoietic angiogenic cells exhibited characteristics of myeloid origins and function whereas OECs demonstrated commitment to the endothelial lineage as had been previously described [36]. For true human EPCs, the cultured population previously coined OECs or late EPCs have been reiterated and are now more commonly identified ECFC [5, 36, 37]. This notion is supported by a growing body of evidence, which suggests ECFCs play a direct and pivotal role in revascularisation of tissue in a range of pathological contexts [38, 39]. Furthermore, there continues to be an emergence of publications, which suggest endogenous neovascularisation is driven in part by tissue-resident ECFCs which do not originate from bone marrow cells [40, 41].

The ECFC exhibits potent intrinsic angiogenic capacity with the ability to assist directly with endothelial repair, instigate *de novo* blood vessel formation and excrete paracrine signals to promote and enrich vascular repair [42–44]. ECFCs are distinguished by the positivity for the cell surface markers CD31, CD146, VEGFR2, VE-Cadherin and dependent on in vitro clonal expansion, CD34. ECFCs must also be negative for hematopoietic markers CD45 and CD14 [44–46]. In 2013, Patel et al. demonstrated the capability to readily isolated large quantities of tissue-resident human ECFCs from term placentas [47]. These ECFCs have the capacity for long term in vitro culture, as well as engraftment and paracrine actions when introduced into host ischaemic tissue [47]. ECFCs display potent intrinsic capabilities to repair damaged endothelium and form tubular structures in vivo, regardless of being freshly isolated or cultured [47].

This much needed demarcation of ECFCs and MACs stems from compiling evidence to suggest in humans at least, that ECFCs derived from bone marrow may not be the only source for endothelial progenitors. Vascular progenitors have been proposed to reside in various organ beds and perform organ-specific functions [48]. The endothelial lining of blood vessels, not limited to a single organ of origin, has been proposed as a source for ECFCs. This theory is supported by the isolation of ECFCs from umbilical veins, aortas, placenta and white adipose tissue [47, 49, 50]. Patel et al. and Shafiee et al. prospectively isolated ECFC from the vasculature of the human term placenta, demonstrating the unique cell surface marker expression and bipotential differentiation capacity of ECFC amongst a novel endothelial hierarchy via flow cytometry and in vitro cell culture methodologies [47, 51]. However, the lack of appropriate tools for in vivo lineage tracing limits the formal demonstration of stemness in these categories of EC. A movement towards understanding the possible phenotypic and functional differences between organ-specific isolation of ECFCs and their endothelial progeny is of great interest for therapeutic purposes [3, 52–55]. The field should consider the pitfalls and complications that may arise in attempting to categorise/define ECFCs in this organ-specific phenomenon.

Therapeutically, the notion that EPCs may be used as a biomarker or possibly treat cardiovascular complications, rheumatoid arthritis, lupus or systemic sclerosis has been documented [56]; however, studying their niche of origin and implementing them for regenerative therapy has had its hurdles [57]. Since the notion that EPCs may be capable of instigating adult vasculogenesis [17], many have tried to isolate EPCs under varied criteria. Arguments surrounding technical isolation and management (cell surface markers, tissue of origin, cell culture conditions) have emerged; however, under the classification outlined by Medina et al. to isolate ECFCs and not EPCs, markers CD31+, CD105+, CD146+, CD45− and CD14− should be adhered to whilst these cells also must be able to form tubes in vivo and in vitro. EPCs have typically been isolated from peripheral or umbilical cord blood for the treatment of human ischaemic disease. However, very few clinical trials have resulted in significant positive outcomes nor have many been designed with placebo arms, let alone been able to validate in vivo vascular integration. Thus, although it has been established that at minimum, EPCs migrate to distressed tissues and indirectly promote vascular regeneration, the full outcome of the efficacy of EPC in treating ischaemic disease in humans has yet been entirely elucidated. Keighron et al. extensively reviewed the use of human EPCs in clinical trials [58]. With new guidelines, various preclinical trials are underway using ECFCs as a cell therapy agent. In reported studies, efficacy and benefit have been observed in 89 % of experiments conducted. Although promising pre-clinical data, no clinical trial has been completed in humans using ECFC [58, 59]. As the field moves towards further harmonising markers and efficaciously implementing the use of ECFCs for regenerative vascular therapy, additional data will come to light if ECFCs pose true therapeutic advantage over current EPC therapy in human patients.

## Defining murine endothelial progenitor cells

The body’s endothelium is heterogeneous and is transcriptionally diverse to perform tissue-specific functions [60]. This, however, accentuates the difficulty of defining, isolating and understanding endothelium in its entirety. Hematopoietic, skin, skeletal muscle and intestinal stem cell niches have been interrogated to great success within recent years [61–64]. However, a consensus on murine EPCs defined through the minimum three criteria required for stringent scrutiny has yet been agreed upon as a surrogate for ECFC research [5, 19]. In part, diversification within the field can be attributed to the vast array of existing experimental models in which ECs can be interrogated in response to various injuries and disease states. Moreover, the quest to find circulating murine EPCs has been an arduous task, and previous isolation attempts have uncovered extremely rare occurrences, if any, of these cells making murine models poor surrogates to study human circulating ECFCs [65].

As we will highlight below, a handful of teams have studied high proliferative capacity and regenerative EPCs in a suite of organ beds. One should consider that traditional methodology may ignore quiescent EPC populations that may be hidden and unbiased scrutiny to re-define a true phenotypic EPC signature is required. Furthermore, stem/progenitor populations are sometimes devoid or lack expression of markers to the lineage that cell pertains before differentiation. (i.e. murine HSCs: CD34^lo/-^) [66]. Lukowski et al. have recently shown that in single-cell sequencing of murine aorta, progenitor cells lie within a CD31^lo/-^ fraction of the *Cdh5* lineage-traced endothelial compartment [67]. This brings into question whether cells pertaining to a specific lineage can be exclusively isolated based on the positivity for a single marker alone. A recent unbiased whole tissue aortic single-cell RNA sequencing (scRNAseq) study revealed multiple endothelial subsets that exist in the aorta with distinct functions outside the domain of vessel subtype [68]. Transcriptomic expression of CD31 was notably varied between the populations posing the question, is positive selection of VECAD+, CD31+ and the discarding of lineage cocktail + cells sufficient to study murine EPCs. Similar judgement could also be considered for the isolation of ECFCs. These questions bring to light complications the EPC field has stumbled upon in the search for a molecular and functional definition of the tissue-resident murine EPC.

## Identifying endothelial progenitor cells

*Bona fide* EPCs were brought into question after other groups demonstrated putative EPCs engraft into peri-endothelial sites and their expression profiles resembled hematopoietic stem and progenitor cells [33, 35, 69]. Since, various techniques have emerged, evolved and come to define true EPCs. A suite of strict criteria encompassing experimental techniques and functional assays are essential to de-convolute findings. These techniques span combinations of in vivo, ex vivo and in vitro techniques.

Foremost, a stem or progenitor cell must possess the ability for clonal expansion, be able to self-renew and give rise to differentiated cell types [70]. Somatic stem cells can either be multi-potent (giving rise to differentiated cells of multiple lineages) or unipotent (governing differentiation of a single-cell lineage). Progenitor cells possess the immense capacity for self-renewal yet display commitment to a single lineage. To interrogate progenitor cell populations, clonal self-renewal and the contribution of the stem cell population to differentiated progeny are analysed via in vivo fate mapping. This in combination with serial transplantation of progenitor cell populations into multiple hosts demonstrates rigid clonal self-renewal characteristics. To examine progenitor cell populations in vitro techniques are commonly employed to analyse the progression of cellular events preceding terminal differentiation. Rigorous scrutiny of EPCs using combinations of molecular (transcriptomic, proteomic, metabolomic) and functional (morphological, self-renewal, vasculogenic potential) analyses provides increasingly complete definitions of the origins of the adult endothelial lineage. A pioneering study by Purhonen et al. rigorously scrutinised the origins of bone marrow-derived circulating EPCs [18]. Utilising lineage tracing models, parabiosed mice and high-resolution confocal microscopy, it was found that all bone marrow-derived cells in the tumour microenvironment were perivascular and not endothelial. This study, amongst others, set the standard for defining murine EPCs. Herein we present a consummation of study and research techniques utilised to discriminate previously described tissue-resident EPC populations.

## Characterising murine EPCs

### Phenotypic isolation and in vitro colony formation assays

Colony formation assays are a conserved bioassay across various fields testing the self-renewal of progenitors. In 2012, Fang et al. published findings that stem cells giving rise to an endothelial lineage expressed the phenotype CD31 + CD105 + Sca1 + CD117 + Lin- and could be isolated from murine organ beds at differing frequencies [71]. CD117 (c-Kit) was used to define the EPC endothelial subset in this study. Fang et al. demonstrated that their initial ECFCs isolated from enzymatically digested tissues exhibited the phenotype CD31 + CD105 + Lin-. These cells were routinely recovered from lung vasculature utilising immunomagnetic lineage bead depletion, consequently allowing for isolation of colony-forming cells at a rate of 1.5:100 of isolated ECs. This clonal growth pattern demonstrated typical progenitor cell characteristics in vitro. EC monolayers were immunostained and a subset of cells notably expressed CD117, a marker commonly elevated in tumour settings, but also associated with hematopoietic and other stem cell populations [72–75]. To validate whether CD117 cell surface expression enriched for EPCs, fluorescence-activated cell sorting of lung ECs (CD31 + CD105 + Lin-) enriched or depleted for CD117 were plated. Colony-forming cells were observed at a frequency tenfold lower in CD117-depleted ECs. Furthermore, the frequency of clonal expansion post-single-cell sorting was observed to be 0.6%, demonstrating within the CD117 + -enriched population lay a functionally phenotypic progenitor cell population. It was noted that CD117 + ECs were prevalent in lungs, liver and kidneys at frequencies of 39, 18 and 2%, respectively. We condense the specifics of each group in terms of identification, organ of origin and description of a differentiation hierarchy in Table 1.

**Table 1:** Identifying murine endothelial progenitor cells (EPCs)

Group	Identification markers	Organ source	Hierarchy	Pathological implications
Fang et al.	CD31 + CD105 + Sca1 + CD117 + Lin-	Lungs, liver, kidneys	No	Neovascularisation in B16 melanoma
Naito et al.	CD31 + CD45-Vecad + SP	Lungs, liver, hindlimb muscle, heart	No	Vascular regeneration following ischaemic injury
Yu et al.	CD31 + CD105 + Sca1 + Lin-Procr+	Mammary glands, Retina	No	Tumour vascularisation and fibrotic diseases
Wakabayashi et al.	CD31 + Vecad + CD45-CD200 + CD157+	Lung, hindlimb muscle, heart, retina, skin, brain, aorta	Yes	Peripheral vessel regeneration and rescue of haemophilia
Patel et al.	Vecad + CD34 + Lin-CD31^lo^ VEGFR2^lo^	Aorta, lung, skin	Yes	Neovascularisation in tumour, wound healing

Various groups have established phenotypic signatures for murine EPCs in hopes to mimic and gather a greater understanding of human endothelial colony-forming cells (ECFCs). Inclusion and exclusion of markers have been used to define murine EPCs in a suite of organ beds, some of which govern a maturation hierarchy of endothelial differentiation. Understanding how these EPCs function in homeostasis as well as pathological contexts offers vast potential for vascular implicated diseases

*SP* side population, *Vecad* vascular endothelial cadherin, *Lin* lineage cocktail

In 2012, Naito et al. utilised a method of in vivo Hoechst staining to identify and characterise vessel-resident progenitor cells [65]. Hoechst 33,342 dye is readily taken up by living cells where it binds to DNA. Previous studies have reported bone marrow hematopoietic stem cells efflux the dye and thus the positive selection of Hoechst^lo^ cells is progenitor/stem in nature. The in vivo injection of Hoechst 33,342 has been used to explore stem/progenitor niches, specifically exploring muscle, skin, heart, lung, mammary gland and testis stem cell niches [76]. It was proposed that resident EC stem/progenitor cells residing within pre-existing vessel networks could be elucidated within the side population (SP) [Hoechst^lo^], whereas the larger endothelial main population (MP) was considered not to be of stem/progenitor nature. Endothelial SP cells sourced from hindlimb muscles were first identified through flow cytometry of CD31 + CD45- fractions, of which they formed 1.15 ± 0.14% of cells compared to the remaining MP. This rare population was scrutinised in vitro for single-cell colony-forming potential on OP9 stromal cells which has been previously used as a feeder layer for expansion of stem cell populations [77, 78]. EC-SP cells were able to be clonally expanded in vitro from various organ beds. A limiting dilution assay of EC-SP and EC-MP highlighted that EC-SP had colony-forming capacity 10 times that of the EC-MP cells.

In a subsequent study from the same group, Wakabayashi et al. pursued their initial EC-SP findings to identify CD157 as a marker for tissue-resident EPCs based on transcriptome analysis [79]. In flow cytometry and immunostaining of various organ beds, it was found that CD157 + CD31 + CD45- ECs could be isolated at differing frequencies between the lung, limb muscle, heart, retina, skin and brain. Immunostaining of the portal vein, hepatic venules, vena cava and aorta confirmed the presence of CD157 + ECs in varying proportions. To validate clonal expansion potential of CD157 + CD200 + ECs in vitro, ECs from the organ beds were plated on OP9 feeder cells or laminin-coated dishes. CD157 + CD200 + ECs plated individually had far greater proliferative potential than CD157-CD200 + or CD157-CD200- ECs. CD157 + CD200 + ECs that gave rise to > 2000 progeny after 14 days of growth and were considered as high proliferative potential (HPP) murine ECFCs. CD157 + HPP murine ECFCs were observed from multiple organ beds.

In 2016, Yu et al. identified protein C receptor (Procr)-expressing ECs at a prevalence of 4% of total ECs (CD31 + CD105 + Lin-) in 8-week-old C57BL/6 mammary glands [80]. It was reported that Procr + cells isolated from mammary vasculature had greater in vitro colony-forming potential, roughly 20 times that of Procr- cells and could be serially passaged to at least 10 passages whereas Procr- cells could only be passaged 3 times.

In 2016, Patel et al. demonstrated the presence of a vessel-resident EPC population present in various tissue beds [81]. An endothelial population was characterised by expression of CD34 and absence of lineage cocktail (CD3, CD11b, CD45, Gr-1 and Ter-119). These cells were CD144+ (VE-Cadherin) and within this VE-Cadherin + CD34 + Lin- population, differing expression levels of CD31 and VEGFR2 allowed for the delineation of an endothelial hierarchy, which differentiated in a maturation process. Three populations were termed endovascular progenitors (*EVP* - CD31^low^/VEGFR2^low^), transit amplifying cells (*TA* - CD31^int^/VEGFR2^low^) and definitive differentiated ECs (*D* - CD31^hi^/VEGFR2^hi^). EVPs were found to have self-renewal capacity from aortic and subcutaneous tumour tissue whereas TA and D cells did not. Endothelial colony formation was conducted and tumour EVPs exhibited 1:100 colony formation capacity, whereas aortic EVPs exhibited 3:100 colony formation capacity [81].

The findings from these four labs demonstrate four uniquely isolated EPC populations, which share some degree of marker homogeneity. These markers were used to isolate EPCs from differing organ beds at differing frequencies (Table 1). It must be considered that alternative means to culture the murine cells, both in terms of basement membrane and supplemental media differed between groups and is refined in Table 2, however, the colony formation potential was somewhat conserved. Although the single-cell assay is required to demonstrate true progenitor/stem capacity, Fang et al. noted that single GFP + CD31 + CD105 + Lin- colony-forming cells formed EC monolayers more effectively when co-cultured with other GFP- stromal cells, suggesting paracrine signalling is a key component for the sustained outgrowth of murine EPCs.

**Table 2:** Assays identifying murine endothelial progenitor cells (EPCs)

Group	Self-renewal capacity and in vitro colony-forming potential	Vasculogenic potential	Plasticity	Lineage tracing/murine models
Fang et al.	Single cells able to propagate for 8–15 passages and give rise to an experimental termination of 1 × 10^8^ progeny Methylcellulose of Gelatin-coated base: 0.6–1.5% Colony-forming potential Culture: IMDM, FCS, L-glutamine, BSA, 2-ME, rh Transferrin, rh Insulin, rm VEGF, rm bFGF, rm EGF	Quaternary transplantation of ECs in B16F0 melanoma	None described	LacZ-β-gal reporter: VEGFR2 and TIE-2. C57BL/6-Tg(*Actb*-eGFP)
Naito et al.	Single cell capacity to give numerous colonies—confirmed by IHC and Flow cytometry. OP9 base: 1.2±0.5% CFP from EC-SP Culture: RPMI-1640, FCS, 2-ME	Hindlimb ischaemia demonstrated EC-SP having 3.5× greater in vivo replicative capacity than EC-MP	None described	*Cdh5-Cre-ERT2* mice Flox-CAT-eGFP
Yu et al.	Procr + ECs could propagate up to 10 passages and give rise to 1 × 10^8^ progeny at experimental termination. Procr- ECs could not propagate past 4 passages. Methylcellulose or Fibronectin base: Colony-forming potential not distinguished numerically, but observed. Culture: IMDM, FCS, L-glutamine, BSA, 2-ME, Glutamax, ITS-X (rh Insulin and transferrin), rm VEGFa, rm bFGF	Freshly isolated GFP + Procr + ECs had a much greater capacity for matrigel plug engraftment as well as pubertal fat pad engraftment, collected 4 weeks post-inoculation. GFP + Procr + ECs isolated, cultured and then injected into hind skin, and fat pads consistently generated functional blood vessels. Limiting dilution of tamoxifen illustrated clone size expansion at 2, 7, 14, 60 and 300+ days using Procr-Cre Procr + injection post-hindlimb ischaemia increased perfusion approximately 70% greater than Procr-injected cells	Yes – observed in mammary glands using Procr and Cdh5 lineage tracing models. Procr model used for skin and retina. Pericyte markers utilised: NG2, Desmin, αSMA, PDGFRb	*Procr-CreERT2-tdTomato/+* *Cdh5-CreERT2; R26* ^*mTmG/+*^ C57BL/6-Tg(*Actb*-eGFP)
Wakabayashi et al.	Single cell capacity to give rise to numerous colonies – confirmed by IF. OP9 feeder base or laminin-coated plates. Multiple organ beds of CD157 + ECs able to proliferate in vitro. Colony-forming potential diverse between tissue beds. Culture: RPMI-1640, FCS, 2-ME, VEGF	Serial transplantation of EC-SP for multiple generations into recipient mice (GFP + CD31 + Vecad + CD45- SP) EC-SP CD157 + CD200 + transplantation post-MCT + Radiation. 20,000 cells provided effective functional revascularisation. Follow-up experiment whereby a single cell was transplanted gave rise to functional liver vasculature 20,000 cells injected sufficient to restore levels of factor VIII secretion from endothelium post-transplant	None described	*Bmx(PAC)-CreERT2/Flox-CAT-eGFP* *Cdh5(BAC)-CreERT2/Flox-CAT-eGFP* C57BL/6-Tg(CAG-eGFP)
Patel et al.	GFP + EVP populations isolated from B16 melanoma and aortic tissues plated on matrigel at limiting dilutions generate colonies whereas progeny do not. Colony-forming potential diverse between aortic and tumour endothelium Culture: EGM-2, FCS	Co-transplantation of EVPs with B16 melanoma cells into new hosts and recovered 7 days after co-transplantation Co-transplantation with matrigel plugs and only EVPs contain capacity to persist and form vascular structures	None described	*Sox18-creERt2/ROSA-YFP* *Cdh5-creERt2/ROSA-YFP* C57BL/6-Tg(CAG-eGFP)

By definition, murine EPCs must (1) have the ability to self-renew, (2) form blood vessels and (3) constitute the intimal layer as well as (4) having the capacity to be serially transplanted to form/incorporate into host vasculature. Herein, we highlight the conditions under which each group scrutinised their defined EPC in culture for self-renewal potential. Moreover, we highlight the transplant experiments each group conducted to demonstrate point 4. We also summarise the lineage tracing models utilised by each group and comment on their observation of endothelial to mesenchymal transition

*2-ME* 2-mercaptoethanol,* bFGF* basic fibroblast growth factor, *BSA* bovine serum albumin, *EC-MP* endothelial main population, *EC-SP* endothelial side population, *EGF* epidermal growth factor, *EGM-2* endothelial growth medium 2, *FCS* foetal calf serum, *IF* immunofluorescence, *IHC* immunohistochemistry, *IMDM* Iscove’s modified Dulbecco’s medium, *MCT* monocrotaline, *VEGF* vascular endothelial growth factor

### In vivo self-renewal and vasculogenic potential 

To robustly conclude a phenotypic population to be of EPC nature, they must self-renew in vivo and form functional blood vessels highlighted in Table 2. The studies highlighted have embarked to demonstrate these characteristics in a variety of settings. Fang et al. published findings in B16F0 melanomas as well as matrigel plugs to demonstrate the importance of CD117 + ECs in the development of neovessels [71]. To analyse the self-renewal potential of the ECs in a pathological context, serial transplantation of GFP + CD31 + CD105 + ECs using C57BL/6-Tg(*Actb*-eGFP) mice was conducted. Authors were able to successfully generate GFP + blood vessels in quaternary transplanted tumours. Additional work by Naito et al. took to understand the kinetics of the EC-SP in vivo [65]. A hindlimb ischaemia (femoral artery occlusion) model was employed to study these populations, where it was found that the EC-SP expanded 3.5-fold (4.03 ± 1.44%) and returned to a steady state 2 weeks post-ischaemic induction. This study demonstrated that endothelial stem/progenitor cells had the capacity to respond to angiogenic stimuli, elicit vascular regeneration and reperfuse ischaemic tissues in vivo. Procr + EPCs were studied in the context of mammary tissues in vivo for their vascularisation capacity with the assistance of C57BL/6-Tg(*Actb*-eGFP) reporter mice [80]. Procr + ECs were shown to form colonies from single cells, be subsequently transplanted into mammary fat pads after colony expansion and form functional vessels connected to circulation. To show that Procr was a marker of vascular progenitors, Procr + ECs and Procr- ECs were injected separately into pubertal recipients (3-week-old) and permitted to develop for 4 weeks. In this post-natal, pubertal vasculogenic event, Procr + ECs were shown to incorporate and assist in the development of vascular networks, whereas Procr- ECs did not elicit vasculogenic potential. Procr + ECs were subsequently tested in culture and showed key endothelial marker expression after the 5th passage, being able to uptake Ac-LDL and express nitric oxide, eNOS and ICAM-1 after IL-1β stimulation [80].

The work of Wakabayashi et al. demonstrated the potential for serial transplantation of EC-SP and incorporation into the liver vasculature whereas EC-MP did not exhibit clonal expansion capabilities past the secondary recipient [79]. The researchers used models of liver injury and C57BL/6-Tg(CAG-eGFP) mice to demonstrate the efficacy of in vivo transplantation. As little as 20,000 CD157 + CD200 + ECs were sufficient to generate portal veins, portal venules, sinusoids, hepatic venules and arteries. In a valiant experiment, single-cell transplantation of a liver CD157 + CD200 + EC into a recipient host post-liver injury generated at least 592 VE-cadherin + CD31 + CD45- daughter cells, which incorporated into portal vein, sinusoids and hepatic venules. This demonstrated that tissue-resident CD157 + CD200 + EPCs could form large and small vessels in vivo and also exhibit self-renewal capacity.

Patel et al. took the approach in line with Fang et al. to study EPCs in vivo in models of B16F0 melanoma and matrigel plug assays [71, 81]. To assess self-renewal, GFP + endothelial hierarchy as demarcated as EVP, TA and D cells was transplanted in matrigel plugs into nude (athymic) mice. It was observed that markedly reduced GFP + cells from C57BL/6-Tg(CAG-eGFP) mice were recovered after 7 days in the TA- and D-transplanted matrigel plugs, whereas EVPs engrafted significantly better and staining of matrigel plugs demonstrated GFP + EVP cells had produced CD31 + VE-Cadherin + vessels. Donovan et al. demonstrated that transplantation of EVPs from mature tumours into new hosts co-transplanted with B16F0 melanoma cells could persist over time, contribute to increased tumour weight and vascularisation, whereas TA and D cell populations could not [82]. These studies illustrate that these EPC populations with different phenotypic markers can be transplanted and passaged in a wide range of tissue beds. As iterated, limiting dilution or more prominently single-cell transplantation is the gold standard of EPC scrutiny in vivo for critiquing stemness and clonal capacity.

## Lineage tracing and plasticity

Lineage tracing using a variety of transgenic mouse models has been widely used in the endothelial field to study vascular systems in a flexible and selective format [83]. Yu et al. were the first group to employ genetic fate mapping (in vivo lineage tracing) of ECs using their *ProcrCreERT2/+;R26mTmG/+* line to label their Procr + ECs of interest. Lineage tracing was conducted in the mammary fat pad of 5-week-old mice using the *Procr-CreERT2;R26-mTmG* to selectively label Procr + ECs after tamoxifen induction [80]. Whole mount immunofluorescence depicted labelling of Procr + ECs 2 days after tamoxifen injection, which clonally expanded (quantified at 7, 14, 60 days after tamoxifen) and contributed to host vasculature for up to 10 months. Notably, clonal expansion of Procr + ECs was also observed in post-pubertal mammary gland; however, the vascular turnover was lesser in the adult mammary gland. Intriguingly, Yu et al. noticed during lineage tracing experiments that pericytes were derived from Procr + EC origins. Immunohistochemical and fluorescence-associated cell sorting of pericytes from endothelial origins were quantified at 2 days, 2 months and 10 months post-tamoxifen. Researchers employed *Procr-CreERT2;R26-Confetti* and *Cdh5-CreERT2;R26-mTmG* lineage tracing models to further validate the de novo formation of pericytes originating from Procr + ECs. These bipotent Procr + EPCs were tested in skin and retinal environments. In vivo clonal expansion of ECs and pericytes was observed and differences in clonal size were attributed to the rate of vascular turnover. The exact lineage tracing models Yu et al. and others utilised to observe endothelial plasticity and behaviour are summarised in Table 2.

Wakabayashi et al. identified *Bmx* as being upregulated in the CD157 + CD200 + EC fraction [79]. Notably, *Bmx* has been validated previously as having a key role in various endothelial settings [84–86]. A *BmxCreERT2/Flox-CAT-EGFP* lineage tracing model was utilised to observe CD157 + EPCs. In a radiation-induced vascular injury model, CD157 + EPCs were labelled 1 week prior to injury. Post-injury, these CD157 + ECs progressively expanded to co-localise with vascular compartments of the liver. Lineage tracing using flow cytometry suggested that CD157 + CD200 + ECs gave rise to CD157-CD200 + and CD157-CD200- ECs after injury. Furthermore, in an analysis of liver maintenance, GFP + cells incorporated into various vascular compartments at 6- and 12-month post-tamoxifen labelling.

Patel et al. uncovered the presence of VECAD + CD34 + CD45- cells migrating into wounds and forming vascular structures independent of bone marrow origins [81]. The waves of EVP cells migrating into granulation tissue and giving rise to TA and D cells were initially studied in a 7-day format. Wounds were analysed using flow cytometry which revealed EVPs were present predominantly at day 1 (D1) and D2 compared to TA and D populations. As time went on, all three populations were present and matured from EVP to TA to D in a maturation process. This time course collection paved the way for subsequent lineage tracing experiments utilising the VE-Cadherin reporter (*Cdh5-cre*^*ERt2*^*/ROSA-YFP*) in healing wounds. The endothelium was labelled with tamoxifen for a single pulse immediately after wounding and it was found that EVP cells were incorporated into granulation tissue at D1, D3 and D5 post-wounding. Flow cytometry and immunofluorescence of D3 and D5 wounds illustrated a continual increase in EVP numbers over time as well as a generation of TA and D cells from EVP origins. Immunostaining uncovered mature YFP + CD34 + vessels expressing CD31 and VEGFR2 becoming ever-present in the centre of the granulation tissue over time in alignment with flow cytometry findings. These results confirmed vascular-resident EVPs mobilised during would healing to initiate neovessel formation in granulation tissue. Authors showed single ECs were not connected to circulation and later formed vascular structures, which were labelled by isolectin after intravenous injection. The researchers would then go onto simulate the same scenario using another transgenic mouse model, the *Sox18-cre*^*ERt2*^*/ROSA-YFP. Sox18* expression is not prevalent in the adult stage, rather the activation of *Sox18* is re-activated within the endothelium stems from pathological stimuli, for example, wound healing and tumour development [87–90]. It was assumed that *Sox18* expression would be upregulated in EVPs once committed to the process of active vessel formation. Results from *Sox18-cre*^*ERt2*^*/ROSA-YFP* mirrored those of *Cdh5-cre*^*ERt2*^*/ROSA-YFP* with YFP + foci present primarily in D1 wounds. The prevalence of the three populations within the endothelial hierarchy shifted towards the D cell phenotype in more mature granulation tissue. Researchers concluded that the majority of EVPs in the wound constitute immature neovessels and more mature vessels emerge with TA and D cells. This is not to discount the possibility that some EVPs in the wound may be isolectin + and reside within mature neovessels.

Another study was conducted in the setting of B16F0 tumours to understand the maturation hierarchy of the endothelium in the tumour [82]. Lineage tracing using the *Cdh5-cre*^*ERt2*^*/ROSA-YFP* and *Sox18-cre*^*ERt2*^*/ROSA-YFP* in a low, single-dose format allowing additional observation of EVPs giving rise to its TA and D cell progeny. This phenomenon was comparable to that of EVPs migrating into granulation tissue in wounds and due to the time course of tumour growth, it was able to be demonstrated that EVPs migrate in waves and give rise to their mature daughter cells. It was also shown that upon multicolour lineage tracing using *Caggsbow* reporter mice, neovessels were from a diverse range of clonal backgrounds, which participated in the formation of arterial, venous, capillary and on occasion lymphatic events. Whether EVPs routinely participate in the formation of lymphatic structures in homeostasis or exclusively pathological events remains undefined. It must be noted, however, that the *Cdh5-cre*^*ERt2*^*/ROSA-YFP* has previously been shown to uniformly penetrate quiescent and active endothelium and detect on occasion lymphatic ECs [91]. These studies highlight the importance and advancement of transgenic technologies. The selective labelling of ECs has enabled vascular researchers to track stem/progenitor cells in vivo and analyse their maturation processes over short and extended periods in a suite of different contexts.

Of great interest for further study is progenitor behaviour, plasticity and bipotency. Understanding how vascular progenitors function, behave and elicit bipotent behaviours may offer invaluable insight into new therapeutic treatments. Recently, endothelial to mesenchymal transition (EndMT) has risen in interest within the domain of vascular biology due to its wide implications in cardiovascular complications [92], wound formation [93], tumour biology [94, 95] and blood brain dysfunction [96]. It has been proposed that endothelial cells may significantly contribute to fibrosis in various conditions and hindering this process may offer means of improving neovascularization and endothelial function in pathological settings. Exactly how EndMT is re-activated in endothelial cells postnatally is reviewed by Piera-Velazquez et al. [97] [98] and Kovacic et al. [99].

It should also be noted there have been studies which observe vascular progenitors outside of the previously mentioned studies that do not contain the four ‘experimental criteria’ to be defined as a true EPC, nonetheless they bring forth promising data to suggest the presence and notion of vascular progenitors. In 2007, Aicher et al. were able to demonstrate c-Kit + CD45- non-bone marrow-derived vascular progenitor cells from intestine and livers were able to instigate neovascularisation in a model of hindlimb ischaemia [100]. Tang et al. were able to illustrate in the arterial adventitia, Sca1 + PDGFRa + stem cells were able to contribute to vascular repair and smooth muscle cell formation [101]. Additionally, in a model of aortic denudation, McDonald et al. demonstrated through rainbow lineage tracing that there was a distinct subpopulation of cells with greater propensity to divide and repopulate the endothelial adventitia [102]. The field is progressing rapidly and with the advent of critical lineage tracing tools allowing for in vivo tracing, the niches, nature and behaviour of these cells are becoming apparent.

## Functional modulation of EPCs and disease models

Utilising EPCs for therapeutics, typically for reperfusion of ischemic tissue, has long held promise within the field. The key issues relating to older findings were whether previously isolated murine EPCs fit the criteria of ECFCs or MACs defined in the human system. The aforementioned studies have searched for murine surrogates to the human ECFC in an attempt to mimic autologous transplant models. Moreover, EPCs are thought to be in almost every organ bed throughout the body and thus are believed to play pivotal roles in a suite of pathological settings requiring further study.

Studying EPCs in disease models and how they form neovessels independent of angiogenesis have been debated for some time. As discussed, Fang et al. utilised matrigel plugs, but also B16F0 melanoma models to study CD117 + EPCs [71]. They noted that the presence of CD117 + ECs was found in human melanoma and breast cancer samples but was not scrutinised as to whether these cells affected survival outcomes. To examine the ability for c-Kit to play a role in melanoma growth, c-Kit^-/-^ mice were employed, which led to defective angiogenesis and reduced the progression of B16F0 melanoma. This transgenic mouse, however, was not lineage restricted to *Cdh5*-expressing cells and may implicate other cell types, such as mast cells, which play an important role in driving angiogenesis [103].

Donovan et al. demonstrated the transplantation of EVPs from mature tumours into new hosts co-transplanted with B16F0 melanoma cells contributed to increased tumour weight [82]. The authors uncovered notch signalling to be upregulated in the EVP fraction. Genetic ablation of notch signalling using *Rbpj*^*fl.*^*/*^*fl.*^*/Cdh5-CreER-RosaYFP* was employed in a model of metastatic melanoma with the propensity to metastasize from subcutaneous sites. Metastatic burden was greatly reduced at secondary sites because of notch signalling knockout in the endothelium suggesting clinically viable targeted therapy against EVP migration into cancerous tissue may offer potential avenues for therapeutic benefit. Interestingly, Anti-VEGF-A treatment did not affect the ability for EVPs to migrate into tumour tissue, possibly suggesting a means for EPCs to work independently of typical angiogenic pathways. This sentiment was initially established in the work of Naito et al. when they noticed that EC-SP contributed to blood vessel formation in tumour environments and that the EC-SP was resistant to traditional anti-angiogenic drug treatment [104].

Patel et al. utilised the *Ragged Oposum* (*Sox18*^*Op/+*^) mouse model to study the depletion of EVPs in the context of wound healing [81]. This mouse model is a dominant negative mutation for *Sox18*, which obviates rescue from *Sox7* or *Sox17*. In this transgenic mouse, it was found that although EVP numbers gradually increased as granulation tissue matured, TA and D cells remained stagnant in the *Sox18*^*Op/+*^ mouse whereas wild-type (WT) granulation tissue vascularised in a typical fashion. Further examination within the wound healing context by Zhao et al. revealed that EVPs possess bipotentiality governed through the co-inhibitory interaction of two transcription factors: *Sox9* and *Rbpj* [93]. Using lineage tracing techniques, they demonstrated that loss of *Sox9* from the endothelium depleted EVPs, whilst enhancing notch signalling and *Rbpj* expression resulting in the maintenance of their endothelial fate and lessening fibrotic burden in the scar tissue. Conversely, deletion of *Rbpj* led to increased *Sox9* expression, pushing EVPs to differentiate towards a mesenchymal fate via endothelial to mesenchymal transition (EndMT), contributing to wound fibrosis and increased scar area. These findings suggest the notion of bipotential differentiation capacity of tissue-resident EVP.

Various laboratories which study angiogenesis routinely use retinal development to study sprouting angiogenesis. Yu et al. explored the role of Procr + EPCs in vascular network formation [80]. It was observed that selective ablation of Procr + ECs (diphtheria toxin transgenic mice) in early post-natal retinal development caused irregular vascular network formation. Additionally, Naito et al. embarked to understand how EC-SP would contribute to revascularisation of ischaemic hindlimb [65]. Results showed EC-SP was effective at expanding in vivo post-transplantation and ischaemic neovascularisation was analysed post-femoral artery occlusion. From the 3000 GFP + EC-SP and GFP + EC-MP cells transplanted, laser Doppler imaging 14 days post-ischaemic event demonstrated that EC-SP had the capacity to reconnect circulation and restore perfusion in the hindlimb whereas EC-MP transplantation had not and necrosis of toes arose.

Wakabayashi et al. took the approach to test the role of CD157 + EPCs in a variety of contexts [79]. When conducting retinal development and vascular reperfusion assays with partial hepatectomy or femoral artery occlusion, the loss of CD157 in CD157-KO mice did not alter outcomes. The authors noted that liver and lymph nodes are known to express abundant levels of factor VIII mRNA and supply circulating factor VIII to assist blood-clotting [105]. Researchers sourced Haemophilia A mice and attempted to transplant CD157 + EPCs as a means to rescue the haemophilic phenotype. It was demonstrated that transplantation of CD157 + CD200 + EPCs greatly increased plasma factor VIII and increased mRNA expression of factor VIII. Tail-clip challenges revealed that CD157 + CD200 + EC transplantation mice stopped bleeding within 5 min compared to 60 min of CD157-CD200 transplantation recipients. This novel study as well as others above highlight the beginnings of how true murine EPCs can be studied for the benefit of finding new ways to target and utilise human ECFCs for therapeutic purposes.

## Distinguishing origins and lineage of EPCs

Whether EPCs derive from vasculature beds of specific tissues or derived primarily from hematopoietic or alternative non-endothelial precursor populations has been the subject of much controversy and studies [5]. Although the notion of tissue-resident EPCs has been shown in humans, one cannot discount the isolation of circulating ECFCs. The isolation of ECFC-like circulating EPCs in the murine system has been particularly difficult and typically not reported upon.

Naito et al. found that during lectin perfusion experiments, 91 % of EC-SP cells were lectin + suggesting that they were at the luminal interface in pre-existing vessels [65]. EC-SP characterised by CD31 + CD45- expression was encountered in various organs and notably not encountered in bone marrow or peripheral blood. Within this study, researchers utilised bone marrow transplantation models in combination with femoral occlusion assays to observe whether GFP + bone marrow-transplanted cells in both neonate and adult systems could release bone marrow-derived cells (characterised by EC-SP phenotype) which would incorporate into hindlimb vasculature. No incorporation of bone marrow-derived cells which were GFP + was identified in the new vasculature, rather quiescent cells in peripheral vessels responded accordingly to ischaemic-induced angiogenic stimuli.

In the follow-up study by Wakabayashi et al. flow cytometry and immunostaining of various organ beds confirmed that CD157 + CD31 + CD45- ECs could be found at differing frequencies between the lung, limb muscle, heart, retina, skin and brain [79]. Immunostaining of the portal vein, hepatic venules, vena cava and aorta demonstrated the presence of CD157 + ECs in varying proportions. Further single-cell RNA analysis of the CD157 + CD200 + EC-SP fraction also validated the upregulation of various endothelial transcripts and minimal presence of potentially contaminating mesenchymal and hematopoietic lineage cells. After it was found that CD157 was not a functional marker for EPCs, but rather an identification and isolation marker, the authors went on to validate possible transcription factors that were responsible for controlling phenotypes observed in CD157 + CD200 + ECs compared to CD157-CD200- ECs. Microanalysis revealed transcription factor expression of *Myc, Fosl2, Atf3 and Sox7* elevated in CD157 + CD200 + ECs.

Although Yu et al. did not conduct bone marrow transplantation studies, they conducted extensive lineage tracing experiments which uncovered bipotentiality of mammary tissue Procr + EPCs [80]. From Procr + EPCs isolated from mammary tissue, RNA sequencing confirmed that the Procr + EPCs exhibited numerous characteristics upregulated resembling vascular development, angiogenesis and EndMT. The findings demonstrated the importance of employing genetic fate mapping and analysing the functionality of EPC behaviour over time which requires further investigation.

Patel et al. noted that in their uncovering of EVPs, they found that the cells were positive for markers Tie2, Sca-1 and CD90.2; however, were negative for c-Kit, mesenchymal marker CD73 and pericyte marker CD146 [81]. EVP, TA and D populations were demonstrated to be present in homeostatic aorta, lung and placental tissue and in pathologically induced wounds and tumours in differing proportions. To confirm the origin of EVPs being vessel resident and not of bone marrow origins, a bone marrow transplant whereby recipients receive reconstituted GFP + bone marrow was performed [106]. Chimeric mice were subjected to dorsal excisional wounding at 8 and 13 weeks and wounds were collected at D1 and D5. It was shown that CD45-CD34 + cells present in the wound were GFP negative including the EVP, TA and D populations. Confocal microscopy confirmed that GFP + cells in the wounds were CD45 + and did not incorporate into CD31 + vessels. Findings, therefore, suggested that cells arising from the bone marrow were not contributing to endothelial populations and neither was the endothelial population contaminated by hematopoietic-derived cells. To uncover the molecular drivers and gene expression levels comparatively between EVP and D cells, uninjured WT mice aortas were processed for RNA sequencing, of which significant differences were found between EVP and D cells. Differentiated ECs exhibited strong upregulation of endothelial-specific markers including CD31, VEGFR2, VWF, notch signalling target genes and absence of hematopoietic markers was observed in all three endothelial populations. In the EVP population, growth factor and cell motility pathways were upregulated as well as endothelial quiescence marker *Il33* and stem cell marker *Sox9*. Patel et al. notably identified *SoxF* family members *Sox7, Sox17* and *Sox18* as being significantly upregulated in the D cell population. Such findings raise questions about tissue heterogeneity, as CD157 + CD200 + EPCs from liver express high levels of *Sox7* in the stem/progenitor fraction.

To further explore the similarities between bulk-RNA sequencing of aorta from the publication of Patel et al. the authors would conduct a follow-up experiment using scRNA-seq to obtain an unbiased picture of the VECAD + CD34 + Lin- endothelial fraction [67, 81]. The researchers found similarities between older sequencing methods; however, much greater cluster definitions from the scRNA-seq analysis. Sequencing also demonstrated that EVPs have unique mitochondrial content and are slower cycling than the D population. Authors would also go on to demonstrate some homogeneity in molecular signatures between EVPs sorted from tumour-derived conditions and the aortic environments, but also highlight the unique signatures obtained in the tumour environment in another publication [82]. RNA sequencing of EVP, TA and D uncovered EVPs to have an upregulation of pathways dedicated to quiescence, stem cell function, mobility and ECM remodelling. D cell populations alternatively expressed key molecular signatures that traditionally represented differentiated endothelium. Additionally, various cytokine signalling pathways, angiocrine and downstream signalling components associated with immune regulation were upregulated in the EVP population prompting interest for further study in this domain [82]. These groups have demonstrated that EPC populations may be phenotypically different between tissues, nonetheless they do share homogeneous functional attributes which can be used when demarcating murine EPCs.

## Endothelial heterogeneity

It has been well established that endothelial populations within different organ beds, location and vessel types are molecularly distinct and heterogeneous [53, 67]. It has become more apparent that endothelial heterogeneity can be utilised as a hallmark for cellular biology, with subpopulations identified across or within specific vessel beds [65, 67, 68, 82, 107]. The work completed by Kalucka et al. in the murine endothelial cells atlas project underpins this idea [107]. Within this project, transcriptomes of single ECs from healthy adult mice were examined across 11 organ beds, with 78 distinct endothelial subclusters identified based on their respective vessel (arterial, venous, capillaries and lymphatics) and organ types. Several interesting observations were made within the project. Firstly, the heterogeneity of ECs was primarily derived from organ type, not the position in the vascular hierarchical tree. This is likely attributed to their organ-specific functions, as EC clusters from various organ beds shared similar transcriptomes based on shared physiological function. As such, ECs from the heart resembled skeletal muscle ECs sharing pathways involved in reducing oxidation and maintaining membrane transport. In addition, functional markers of specialised ECs were identified within the intestines (*Madcam1* vein ECs) and the brain (*Aqp7* capillary ECs), which may be useful uncovering alternative mechanism in their respective organ bed. The notion of organ-specific gene expression heterogeneity, based on their function, in ECs was further supported by the re-analysis of single-cell RNA sequencing of ECs from the Tabula Muris consortium project [108]. Pathway analysis on the most differentially expressed genes in ECs across organ beds demonstrated functionally dictated gene expression profiles in ECs, within a given tissue. Nevertheless, key developmental pathways critical for maintaining endothelial homeostasis such as Wnt, mitogen-activated protein kinase (MAPK) and cytokine receptor interaction pathways were conserved across the endothelium, throughout each organ beds.

The murine endothelial atlas project and the Tabula Muris consortium have significantly expanded the current understanding of EC heterogeneity at the transcriptome level through each specific tissue type. A key finding established from these two datasets was the EC heterogeneity dictated by their respective function, arising from organ/tissue origin. Consequently, further mechanistic insights, by multi-omics analyses (proteomic and phospho-proteomics), are needed to validate and support enriched pathways identified. The work completed by Inverso et al. [109] demonstrated the importance of this through examining the liver endothelium. Specifically, it was demonstrated that 10% of transcribed genes based on single-cell transcriptomics were identified as significantly up- and down-regulated on the post-transcriptional level, additional phospho-proteomic analysis revealed the most immediate insight into EC phenotype and function within the tissue bed as well as at the molecular pathway level. Taken together, these emerging techniques provide powerful and valuable information on endothelial heterogeneity and their respective function, leading to the identification of previously unrecognised endothelial subtypes. Indeed, the combination of multi-omics can be utilised to recognise and define endothelial progenitor populations based on their molecular expression, which may be dictated by their function.

Following their initial functional characterisation of the EVP maturation hierarchy, Lukowski et al. used a data-driven, unbiased approach to study the molecular profiles of the hierarchy via single-cell RNA sequencing [67]. Preparation and subsequent sequencing of sorted mouse aortic endothelium (VE-Cadherin + CD34 + LIN-) displayed two distinct endothelial populations, which had a significant correlation of gene expression with the previously identified EVP and D cells, suggesting that these are representations of the same endothelial populations. Metabolic activity was upregulated within the progenitor cluster demonstrated by a specific increase in the expression of genes involved in mitochondrial activity, validated by tetramethylrhodamine methyl ester perchlorate levels. Classically, activation of mitochondrial metabolism during differentiation was deemed a necessity to fuel the metabolic needs of differentiated cells [110]. However, several components of mitochondrial metabolism and respiration have now been shown to be crucial in maintaining the self-renewal capacity of progenitor populations [110]. Another key idea proposed by Lukowski et al. was the potential for the EVP population cluster to have bipotent capability, being able to give rise to both endothelial and mesenchymal cells. This idea is supported by the homogeneous high expression of well-described mesenchymal cell surface markers such as *Pdgfrα* and transcription factors such as *Sox9* [81]. The ability of endothelial progenitors to give rise to mesenchymal cells was demonstrated by Shafiee et al. [51] where they isolated a specific endothelial fraction of the human term placenta that was VE-Cadherin + CD43 + CD45-CD31_low_. Similar to EVPs, this population also displayed high expression of mesenchymal genes, including *Pdgfrα*, and gave rise to both endothelial and mesenchymal colonies in vitro after long-term culture [51]. PDGFRα has been shown to have implications in angiogenesis and wound healing and has recently emerged as a potential marker for mesenchymal stem and progenitor cells [111, 112]. Santini et al. recently showed in a *PdgfrαH2B- eGFP* mouse model of hindlimb ischaemia that after 7 days, GFP + cells were found to be associated with what appeared to be newly forming vasculature, decreasing in GFP expression likely due to increased endothelial lineage commitment [113]. An additional study during embryonic development demonstrated the crucial role for *Pdgfra* within the endothelium in forming mesenchyme-derived structures, such as cardiac cushions [114]. The resultant depletion of *Pdgfra* in the endothelium during development prevented the correct formation of cardiac ventricles [114]. Taken together, one could suggest that the EVP cluster is potentially a conserved bipotent progenitor cell in the adult endothelium that maintained this exclusive capacity and warrants further functional studies in vivo.

The extent of EC heterogeneity within arterial vessels was further explored by Kalluri et al. through single-cell RNA sequencing [68]. Whole mouse aorta was enzymatically dissociated; however, subsequent sequencing was conducted on unsorted aorta sample. Clustering analysis identified 10 populations, which represented each of the main arterial cell types, including ECs, fibroblasts, vascular smooth muscles and immune populations such as monocytes, macrophages and lymphocytes. Within the endothelial cluster, 3 distinct sub-endothelial populations were identified and displayed the most significant cellular heterogeneity. Gene enrichment analysis of these endothelial populations revealed their unique function and phenotype, with one lymphatic endothelial cluster and 2 other populations that specialised in lipoprotein handling, angiogenesis and extracellular matrix production.

Lukowski et al. and Kalluri et al. studies demonstrated the presence of endothelial heterogeneity amongst the aortic endothelium; however, the proposed molecular function of the identified clusters between these two groups differs significantly [67, 68]. As previously mentioned, Lukowski et al. defined two major population clusters within the aortic endothelium distinguished by the varied expression of stem cell and quiescence related genes. However, the two major vascular endothelial clusters proposed by Kalluri et al. [68] displayed significant heterogeneity in endothelial-specific functions. Reactome pathway enrichment of functional gene sets revealed subpopulation cluster 1 specialises in extracellular matrix production and integrin adhesion, which suggest their role in vascular remodelling and leukocyte interaction. In contrast endothelial cluster two showed high expression of genes related to lipoprotein handling and angiogenic tip cell formation. Moreover, the transcriptional heterogeneity can also identify how these endothelial populations respond to pathological stimuli. The up-regulation of smooth muscle contractile gene signature in ECs cells after exposure to high-fat diet is particularly interesting as EndMT is a common pathway in atherosclerosis genesis and development [115, 116].

The cellular heterogeneity of the vasculature within other major organs was additionally characterised by He et al. through single-cell RNA sequencing [117]. The vascular endothelium of the brain and lungs was marked by a set of transgenic reporter mouse *Cldn5*(BAC)-GFP, and single cells were isolated through fluorescence-activated cell sorting. The constructed expression data defined 6 types of ECs: venous, capillary, arterial and three others. In comparison to the work done within the mouse aorta, He et al. demonstrated distinct heterogeneity between venous, arterial, and capillary networks at an organ level. Furthermore, the additional 3 endothelial clusters could represent the progenitor population proposed by previous studies. However, He et al. did not conduct any pathway or differentiation analysis to further dissect the molecular differences between those endothelial populations.

## Endothelial regeneration

Regenerative medicine techniques to recover cardiac and vascular function are becoming widely accepted as a potential viable cardiovascular disease treatment. Resident EPCs can form new vascular networks through vasculogenic or neovascularisation processes. A vast number of pre-clinical studies have accumulated strong evidence, which demonstrates the robust regenerative potential of EPCs in myocardial infarction (MI), limb ischaemia and wound healing [26, 51, 118]. In addition, changes in EPC molecular signalling pathways can be considered biomarkers for vascular diseases [27, 119–121]. Of particular interest, chronic heart failure because of myocardial infarction has reached epidemic proportions. As such, understanding the origin and clonal proliferative capability of ECs associated with neovascularisation after injury is essential for the advancement of heart failure therapeutics. In 2019, Li et al. demonstrated a single-cell-based gene expression atlas of cardiac-resident ECs and the transcriptional hierarchy which underpins endogenous vascular repair following MI [41]. Their observation that clonal expansion of resident cardiac EC is the predominant mechanism driving neovascularisation further expands on the findings of He et al. in 2017, who showed new cardiac vasculature is formed from pre-existing ECs [118]. Furthermore, the structural integrity of the cardiac vasculature during homeostasis, or following MI, was shown to be maintained by a subset of ECs with progenitor-like function marked by the expression of platelet-derived growth factor B (*Pdgfb*).

Through single-cell RNA sequencing, Li et al. also defined endothelial heterogeneity in homeostatic and injured mouse hearts [41]. Ten cardiac endothelial clusters were characterised with distinct gene expression signatures in homeostasis and MI, with 5 clusters largely constituted by cells from the MI heart. Interestingly, one cluster was composed exclusively of cells as the result of MI. This was the first in-depth characterisation of the molecular profile which demarcate resident heart endothelial heterogeneity and plasticity in response to MI. However, Li et al. did not identify significant changes in EndMT gene signatures or the loss of endothelial-specific markers. This contrasts with a previous report which demonstrated EndMT contributes to the progression of cardiac fibrosis modulated through BMP-7 as well as a recent finding, which suggests that partial EndMT supports cardiac neovascularisation through vessel stabilisation after MI [122, 123].

## Conclusion

**Fig. 1:**
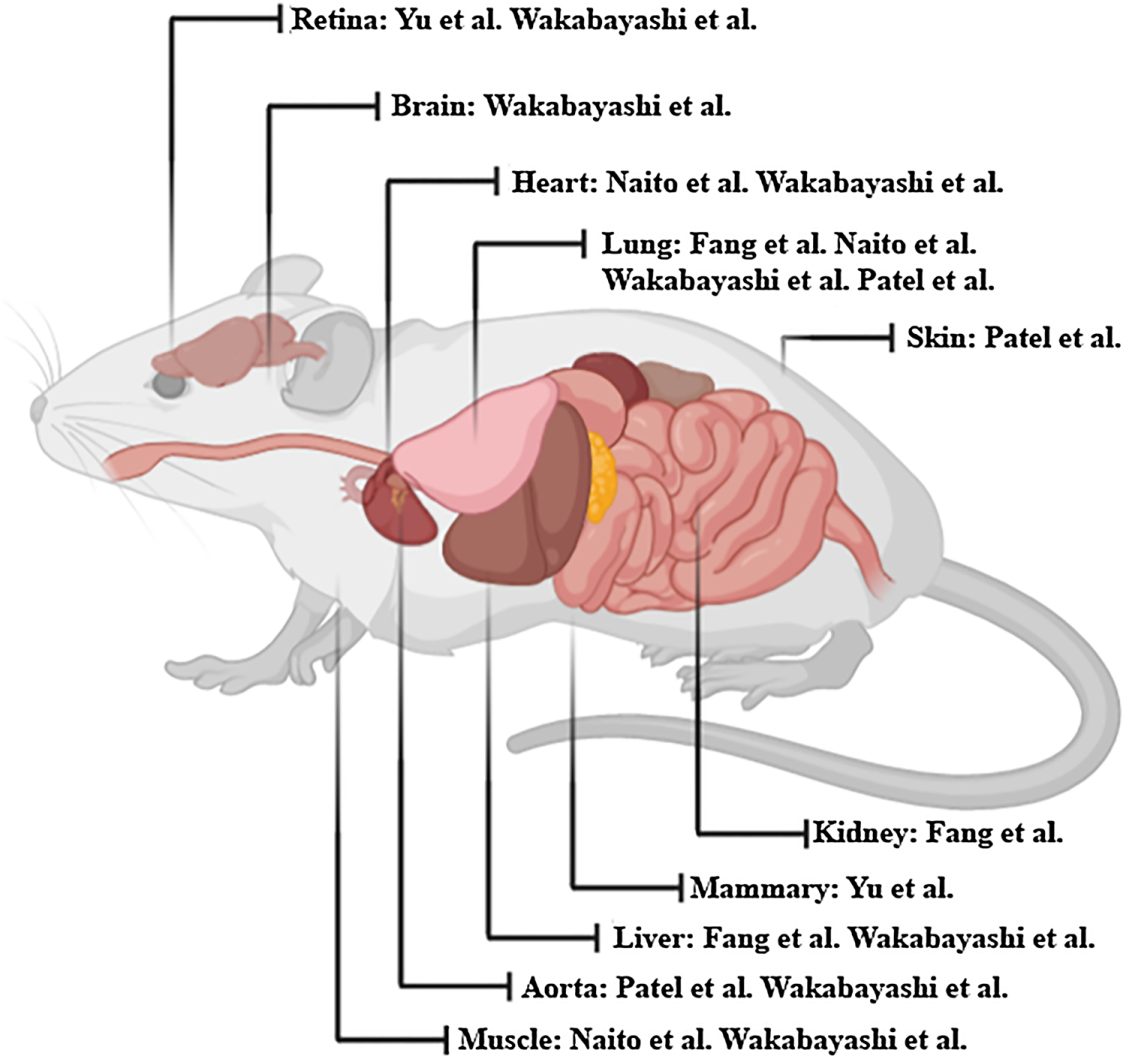
Phenotypic characterisation and isolation of murine endothelial progenitor cells (EPCs). 5 seminal papers have demonstrated that murine endothelial progenitor cells (EPCs) can be isolated and passaged in culture for an extended period. We summarise the groups which have discovered murine EPCs in various organ beds

We have summarised key phenotypic markers, which have been used to isolate EPCs from differing tissue beds (Table 1), numerous studies of which have denoted the presence of an endothelial hierarchy governed by a population with higher self-renewal capacity and greater therapeutic benefit. These seminal papers highlighted have all shown components of the four consensus recommendations, which govern the term EPC in its current iteration [19, 71, 79–81]. This review specifically assess’ EPC populations, which give rise to endothelial progeny, highlighting the methodologies and means of characterising murine EPCs (Fig. 1). However, despite these findings of endothelial heterogeneity and the differences between in vivo studies, they all point to a unique direction; the existence of a tissue-resident EPC. Future studies from here will need to identify common overlaps between previous findings to create a consensus of definition for in vivo EPCs that will further refine our understanding of the unique endothelium.

## Abbreviations

EC: Endothelial cell
ECFC: Endothelial colony-forming cell
ECM: Extracellular matrix
EPC: Endothelial progenitor cell
HPP: High proliferative potential
Lin: Lineage cocktail
MACs: Myeloid angiogenic cells
MAPK: Mitogen-activated protein kinase
MP: Main population
OEC: Outgrowth endothelial cell
scRNAseq: Single-cell RNA sequencing
SP: Side population
VEGF: Vascular endothelial growth factor
VEGFR2: Vascular endothelial growth factor 2
